# CT Features of Colorectal Schwannomas: Differentiation from Gastrointestinal Stromal Tumors

**DOI:** 10.1371/journal.pone.0166377

**Published:** 2016-12-22

**Authors:** Ji Hee Kang, Se Hyung Kim, Young Hoon Kim, Sung Eun Rha, Bo Yun Hur, Joon Koo Han

**Affiliations:** 1 Department of Radiology, Seoul National University Hospital, Seoul, Korea; 2 Department of Radiology, Seoul National University College of Medicine, Seoul, Korea; 3 Department of Radiology, Seoul National University Bundang Hospital, Seongnam, Korea; 4 Department of Radiology, The Catholic University of Korea, Seoul, Korea; 5 Department of Radiology, National Cancer Center, Goyang, Korea; 6 Institute of Radiation Medicine, Seoul National University Medical Research Center, Seoul, Korea; Universidade do Minho, PORTUGAL

## Abstract

**Purpose:**

To find differential CT features of colorectal schwannomas from gastrointestinal stromal tumors (GISTs).

**Materials and Methods:**

CT features of 13 pathologically proven colorectal schwannomas and 21 GISTs were retrospectively reviewed. The following CT items were analyzed: size, longitudinal and transverse location, shape, margin, homogeneity, necrosis, surface ulceration, calcification, degree of attenuation, the presence of enlarged lymph node (LN), and metastasis. Among the features, significant variables were evaluated using univariate statistical tests. The optimal cut-off point of tumor size was obtained by ROC analysis. Binary logistic regression analysis was used to find the most independent CT variables.

**Results:**

Small size, non-rectum location, smooth margin, homogeneous high attenuation without necrosis, and the presence of enlarged LNs were found to be significant variables to differentiate schwannomas from GISTs (P<0.05). The optimized cut-off point for tumor size in distinguishing GISTs from schwannomas was 3.9 cm (AUC = 0.808, sensitivity = 66.7%, specificity = 92.3%, P<0.0001). Binary regression analysis revealed that only non-rectum location remained independent predictor for schwannomas differentiated from GISTs (odds ratio = 31.667, P = 0.001).

**Conclusion:**

Colorectal schwannomas usually located in non-rectum and appear as small subepithelial nodules showing homogeneous high attenuation and smooth margin. Schwannomas exclusively accompany with enlarged LNs.

## Introduction

Schwannomas are rare tumors that develop from Schwann cells. The colon is the least common site for intestinal involvement and the stomach is the most [1, 2]. The exact prevalence of colonic schwannoma is unclear; however, it has been indirectly estimated that schwannomas are significantly less common than gastrointestinal stromal tumors (GISTs) by a ratio of 8–100:1 [3, 4].

Colorectal schwannomas have received limited attention from clinicians as well as radiologists due to their rarity. However, imaging technique advances and increased incidences of routine health check-ups have increased the number of incidentally found colorectal mesenchymal tumors. Most colorectal mesenchymal tumors are GISTs; however, other benign mesenchymal tumors including schwannomas can be found. The exact preoperative differentiation between GIST and schwannoma has clinical significance because colorectal schwannomas are completely benign and totally different from the malignant potential of even small GISTs. However, to the best of our knowledge there have been no reports addressing this issue.

Choi et al. recently reported that gastric schwannomas show distinctive CT features differentiating them from gastric GISTs [5]. Gastric schwannomas appear as a homogeneous low attenuating mass without necrosis and frequently accompany with enlarged lymph nodes (LNs) on CT [5]. We hypothesize that colorectal schwannomas also have similar differential CT features from colorectal GISTs. Therefore, the purpose of this study is to find differential CT findings of colorectal schwannomas from GISTs.

## Materials and Methods

This retrospective study was approved by the institutional review board (IRB) of four hospitals and the requirement for informed consent was waived. The IRB permit number of each hospital is: 1601-033-733 from Seoul National University Hospital, B-1607-355-401 from Seoul National University Bundang Hospital, KC16EIME0016 from The Catholic University of Korea, and NCC2016-0164 from the National Cancer Center.

### Patients

We consecutively collected cases of colorectal schwannoma and GIST from our pathology database from January 2000 to December 2015. Due to the rarity of colorectal schwannomas, seven cases of colorectal schwannomas were additionally obtained from three other institutes (Seoul National University Bundang Hospital, The Catholic University of Korea, and the National Cancer Center). Patient information including demographics, type of surgery, final histopathologic results was provided from other institutes. All CT data from other institutes were anonymized. The following inclusion criteria were used in this study: (a) pathologically proven schwannoma or GIST of the colorectum; (b) lesions with available contrast-enhanced CT images before any treatment. Finally, 34 patients with colorectal schwannomas or GISTs were enrolled: 13 patients with colorectal schwannomas (7 men and 6 women; mean age, 61.8 years; range, 45–76 years) and 21 patients with GISTs (15 men and 6 women; mean age, 60.5 years; range, 37–76 years). All tumors were pathologically confirmed by either surgery (n = 32) or biopsy (n = 2). Type of surgery was recorded for patients who underwent surgery. In cases of GISTs, risk classification according to Miettinen and Lasota was performed [6]. This classification defines three risk groups for colorectal GIST: no risk GIST (≤ 2 cm and exhibiting ≤ 5 mitoses per 50 high-power fields [HPFs]), low risk GIST (2–5 cm and exhibiting ≤ 5 mitoses per 50 HPFs), and high risk GIST (either > 5 cm or exhibiting > 5 mitosis per 50 high-power fields). The institutional review board of our center as well as three other institutes approved this retrospective study. The requirement for informed consent was waived.

### CT Acquisition

The retrospective design of our study required a variety of CT scanners. Most CT scans (28/34, 82.4%) were performed with one of the following 11 MDCT scanners with 4–128 detector-rows: Mx8000 (n = 3) or Brilliance 64 (n = 9) (Philips Medical Systems, Cleveland, OH, USA), LightSpeed Ultra (n = 2), LightSpeed 16 (n = 1), LightSpeed VCT (n = 1), or Optima CT660 (n = 1) (General Electric Medical Systems, Milwaukee, WI, USA), Sensation 10 (n = 1), Sensation 16 (n = 3), Sensation 64 (n = 4), Sensation Definition (n = 1), or Sensation Definition Flash (n = 2) (Siemens Medical Solutions, Forchheim, Germany). The remaining six CT scans were obtained using one of two single-detector CT scanners (Somatom Plus S [n = 2] or Somatom Plus 4 [n = 4], Siemens Medical Systems, Erlangen, Germany). The section thickness was 2.5–8 mm and reconstruction interval was 2–5 mm. Detector configuration, pitch, rotation time, tube voltage and tube current were 0.625–5 mm, 0.89–1.35, 0.5–1 sec, 120 kVp, and 150–250 mAs. Among 34 patients, 2 patients underwent CT colonography (CTC) with fecal tagging using 60 ml of 40% barium solution and 50 ml of Gastrografin. All CT scanning was performed in supine position except for two CTC examinations in which supine and prone position CT were obtained.

All patients received an iodinated contrast agent injection (Ultravist 370, Bayer Schering Pharma, Berlin, Germany) using an automatic power injector at a rate of 3–5 mL/sec and a dose of 1.5 mL/kg. All CT scans in 13 patients with schwannomas were obtained in single portal phase 60–70 seconds after contrast administration while CT scans in 4 patients with GISTs were obtained in both arterial and portal phases. Arterial phase images were scanned at 13–17 seconds after attenuation of the descending thoracic aorta reached 100 Hounsfield units (HU) using a bolus tracking technique.

### Image Analysis

Morphological features and enhancement patterns of the tumors were independently assessed by two radiologists in consensus (K.J.H and K.S.H with 2 and 17 years of experience) on a picture archiving and communication system (PACS) workstation monitor (m-view, INFINITT, Seoul, Korea). The radiologists knew that all patients were diagnosed as having either colorectal schwannomas or GISTs, but were blinded to the exact diagnosis.

The following CT features were analyzed: size (cm), longitudinal and transverse location of the tumor, shape, margin, homogeneity, the presence of necrosis, surface ulceration, calcification, degree of enhancement on each CT phase, the presence of enlarged lymph node (LN), and metastasis. Longitudinal location was divided into cecum, ascending colon, transverse colon, descending colon, sigmoid colon, and rectum while transverse location was divided into endophytic, dumbbell, and exophytic. Tumor shape was evaluated as round or oval and tumor margin was defined as smooth or lobulated. Degrees of enhancement were compared to that of normal back muscle and defined as high-, iso-, or low- attenuation on the arterial and portal phases. LN was considered enlarged when its long diameter exceeded 8 mm.

### Statistical Analysis

Statistical analyses were performed with SPSS 22.0 for Windows (SPSS Inc., Chicago, IL, USA). Chi-square or Fisher’s exact test for categorical variables and the Mann-Whitney U test for continuous variables compared the prevalence of each CT feature between schwannomas and GISTs. A binary logistic regression analyses with a forward LR method were used to assess the most significant CT feature in differentiating schwannomas from GISTs. Receiver operating characteristic (ROC) analysis was performed to find the optimal cut-off value of tumor size. A value of P < 0.05 was considered statistically significant.

## Results

### Clinical and Histologic Findings

All 13 patients with colorectal schwannoma underwent colorectal surgery: ileocecectomy (n = 1), right hemicolectomy (n = 1), left hemicolectomy (n = 3), anterior resection (n = 1), low anterior resection (n = 5), and transanal excision (n = 2). However, 19 of 21 patients with colorectal GISTs underwent surgery: mass excision (n = 4), left hemicolectomy (n = 1), anterior resection (n = 1), low anterior resection (n = 5), ultra-low anterior resection (n = 3), and transanal excision (n = 5). Histopathologic results were obtained through colonoscopic biopsy in the remaining 2 patients with 9.2 cm and 12.3 cm rectal GISTs who received chemotherapy using a molecular targeted agent (imatinib) because the tumors were too large to undergo radical curative R0 resection.

In all 13 patients with schwannoma, lymphoid cuffing was found around the tumor and immunohistochemical results for S-100 protein was positive. In 5 patients with schwannoma, LN hyperplasia in 2–25 retrieved LNs was described in pathologic reports. Among 21 patients with GISTs, 17 had high risk GISTs, 3 low risk GISTs, and 1 had no risk GISTs. All 21 GISTs were positive for c-kit protein. According to the American Joint Committee on Cancer (AJCC) 7^th^ staging system for GIST [7], one patient was T1, eight were T2, seven were T3, and five were T4. LN metastasis was not found in all 19 GIST patients who underwent surgery. Total mesorectal excision was performed in eight patients with GISTs who underwent low (n = 5) or ultra-low (n = 3) anterior resection.

### CT Features

**Fig 1:**
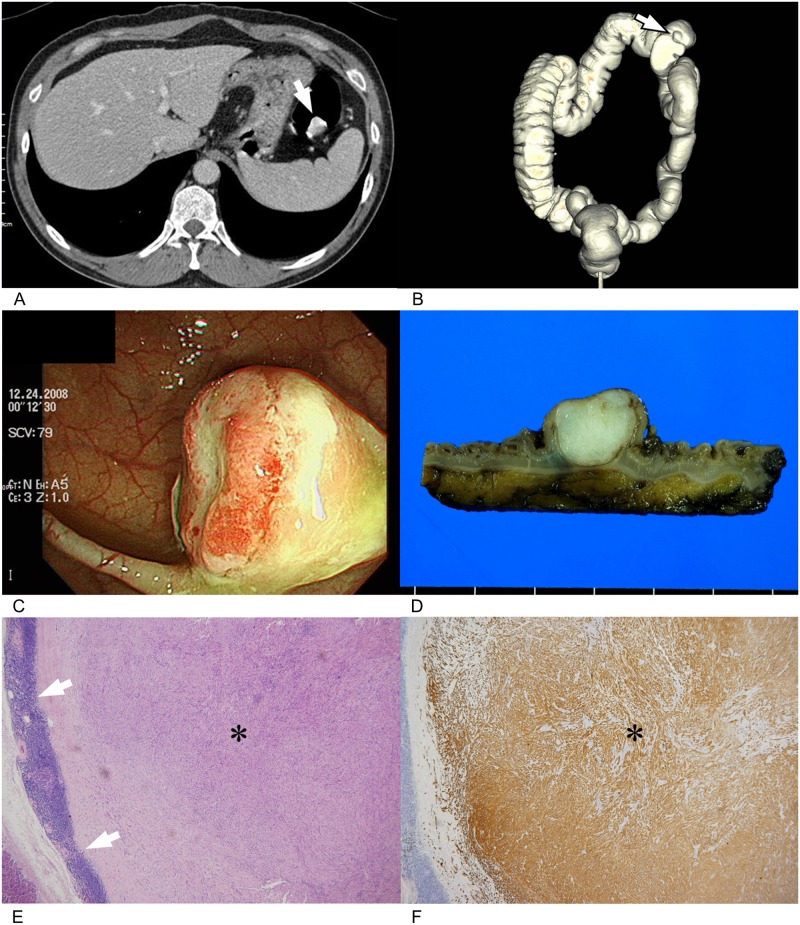
A 45-year-old man with colonic schwannoma. **(A).** On axial (A) images of CT colonography (CTC), a 1.7 cm endophytic lesion (arrow) is seen at the distal transverse colon. The lesion shows homogenous high attenuation compared to back muscle. There were no surface ulceration or calcification in the lesion. **(B).** Opaque (B) images of volume rendering CTC also demonstrate the lesion (arrow). **(C).** Colonoscopy shows an endophytic subepithelial lesion with surface ulceration. **(D).** A photograph of cut surface of gross specimen obtained after left hemicolectomy shows a well-defined endophytic subepithelial nodule in the colon. **(E).** On low-power field microscopic examination (H&E, x10), spindle cell tumor (*) is surrounded by characteristic peripheral lymphoid cuffs (arrows). **(F).** Tumor cells (*) are diffuse and strongly S-100 protein positive on immunohistochemistry and confirmed as colonic schwannoma involving the submucosa and proper muscle. Reactive hyperplasia was noted at 16 lymph nodes in the resected specimen (not shown). H&E = hematoxylin and eosin.

**Fig 2:**
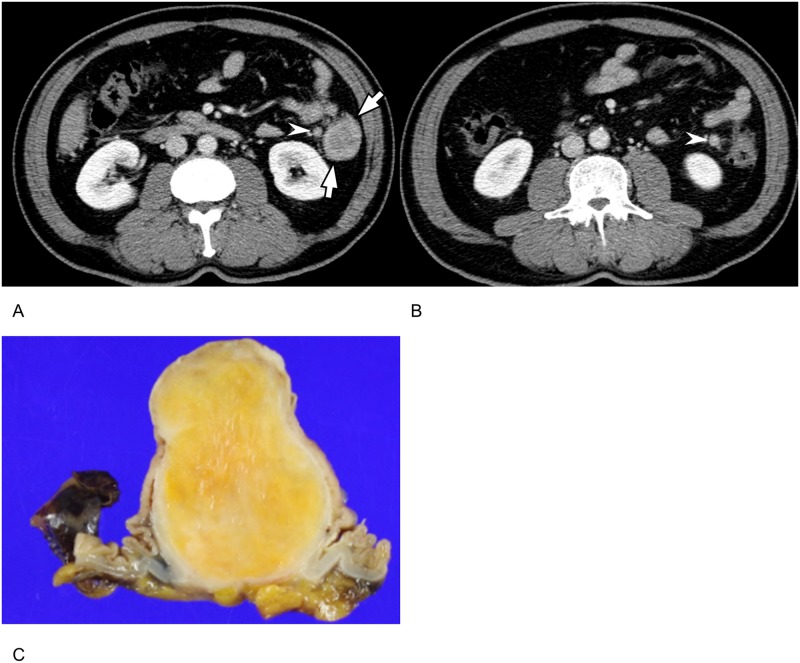
A 58-year-old man with colonic schwannoma. **(A), (B).** On portal phase axial CT images, a 3.9 cm round mass (arrow, A) is noted at the descending colon. The mass shows homogeneously higher attenuation than back muscle with smooth margin. Note the two enlarged enhancing lymph nodes (arrowheads) at pericolic area. **(C).** A photograph of the cut surface of gross specimen obtained after left hemicolectomy shows a well-defined endophytic subepithelial mass in the colon. Tumor cells are diffuse and strongly S-100 protein positive on immunohistochemistry and confirmed as colonic schwannoma involving the submucosa and proper muscle (not shown).

**Fig 3:**
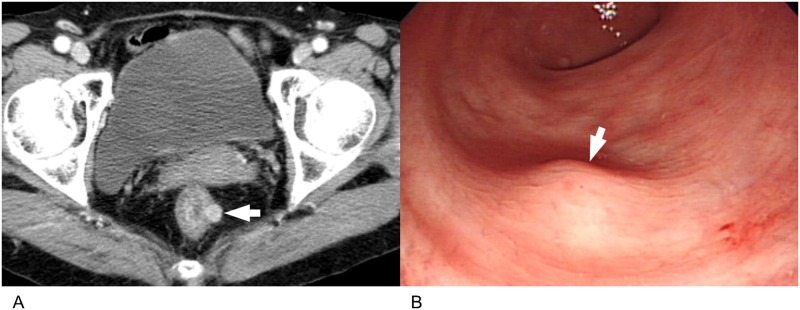
A 76-year-old woman with rectal schwannoma. **(A).** On portal phase axial CT image, a 1.1 cm round nodule (arrow) is seen at the rectum. The lesion shows homogeneously higher attenuation than back muscle with smooth margin. **(B).** Colonoscopy shows a smooth elevated subepithelial lesion (arrow) in the rectum.

**Fig 4:**
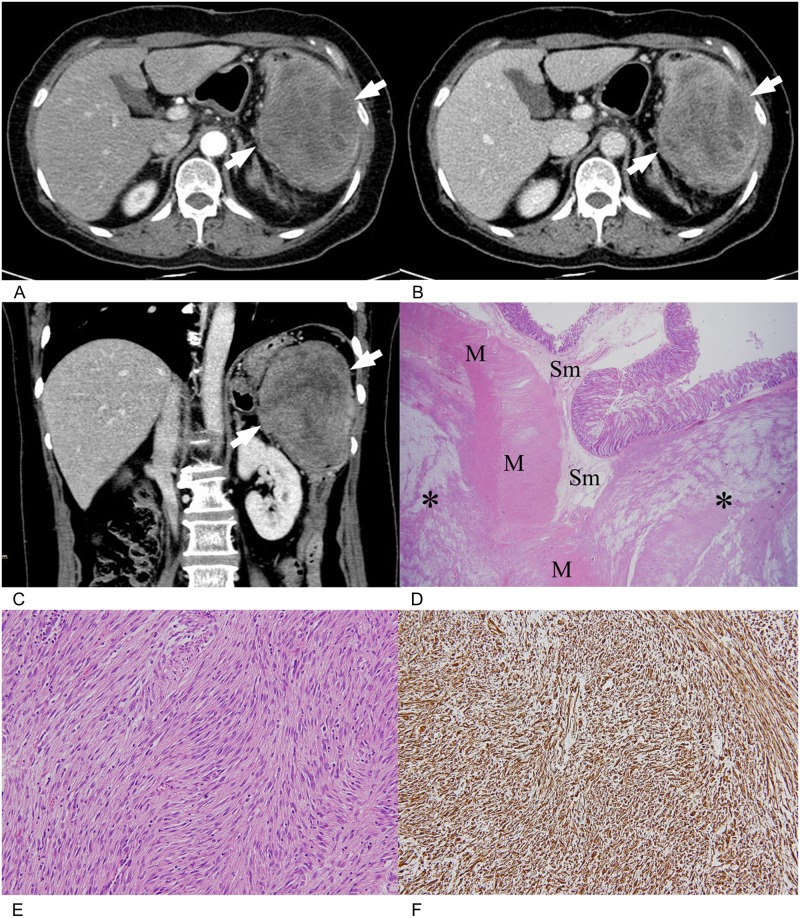
A 57-year-old woman with colonic gastrointestinal stromal tumor (GIST). **(A), (B).** On arterial (A) and portal (B) axial CT images show a 10 cm large heterogeneous ovoid mass (arrows) in the descending colon. The mass shows high attenuation than back muscle on both phases. **(C).** Coronal CT image also demonstrates a large heterogenous mass (arrows) in the descending colon. **(D).** Left hemicolectomy was performed and histopathology (H&E, x10) revealed spindle cell type tumor (*) invading submucosa (Sm) and proper muscle (M). **(E).** High-power field microscopic photograph (H&E, x100) shows a highly cellular tumor composed of broad bundles of elongated cells. Mitotic count of this tumor was 15/50 HPFs. **(F).** Tumor cells are diffusely c-kit positive on immunohistochemistry. Therefore, GIST with high risk of malignancy was finally diagnosed. H&E = hematoxylin and eosin. HPF = high-power field.

**Fig 5:**
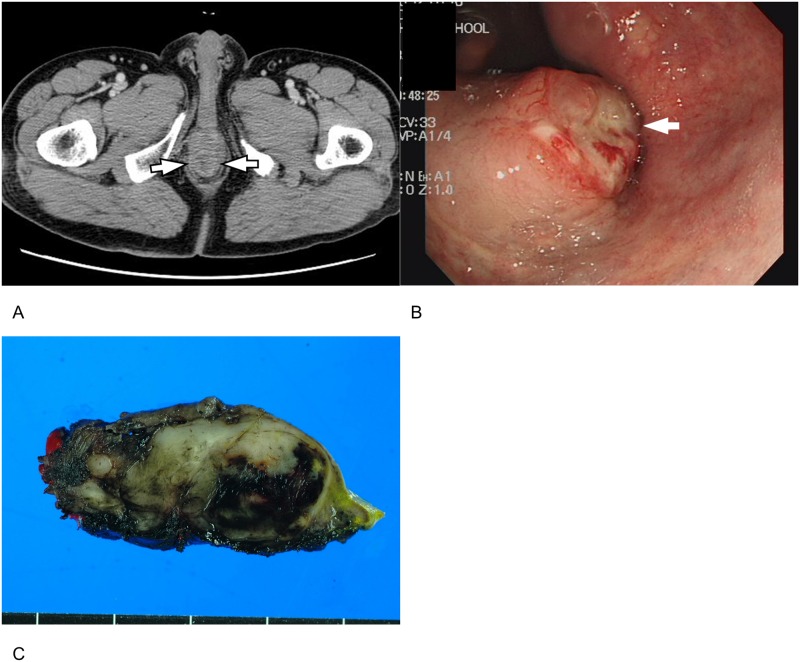
A 73-year-old man with rectal gastrointestinal stromal tumor (GIST). **(A).** Portal phase axial CT image demonstrate a 1.8 cm round nodule (arrows) at the distal rectum. The lesion shows heterogenous iso-attenuation. **(B).** Colonoscopy shows an endophytic subepithelial lesion (arrow) with surface ulceration in the distal rectum. **(C).** Transanal excision was performed and final histopathology confirmed rectal GIST with a high risk of malignancy due to high mitotic count (12/50 HPFs) (not shown). HPF = high-power field.

Table 1 lists the results of univariate analysis between schwannomas and GISTs. Mean age and sex distribution were not significantly different between the two groups. However, mean size (6.3 cm) of GISTs were significantly larger than that (2.4 cm) of schwannomas (P = 0.002). The optimized cut-off value for tumor size to differentiate GISTs from schwannomas was 3.9 cm (AUC = 0.808, sensitivity = 66.7% [14/21], specificity = 92.3% [12/13], P<0.0001). Most (19/21, 90.5%) GISTs were located in the rectum, while most (10/13, 76.9%) schwannomas were located in non-rectum: ascending colon in two, transverse colon in two, descending colon in one, and sigmoid colon in five (P<0.0001). Most schwannomas (11/13, 84.6%) had a smooth margin (Figs 1–3), while half of GISTs (11/21, 52.4%) had a lobulated margin (P = 0.034). Except for one, all schwannoma (12/13, 92.3%) showed homogeneous attenuation without necrosis (Figs 1–3) while half of GISTs (10/21, 47.6%) showed heterogeneous attenuation with necrosis (P = 0.017) (Figs 4 and 5). In terms of degree of enhancement, most (11/13, 84.6%) schwannomas showed high attenuation compared to back muscle, while two-thirds (15/21, 71.4%) of GISTs showed iso-attenuation on portal phase (P = 0.002). LN enlargement was exclusively found in four schwannomas (4/13, 30.8%) (P = 0.015) (Fig 2). Calcification (5/21, 23.8%) and liver metastasis (2/21, 9.5%) were exclusively found in GISTs, without statistically significant differences (P = 0.073 and 0.374, respectively). Two patients with metastatic GISTs were all high risk GISTs. Representative examples are presented in Figs 1–5.

**Table 1:** Results of univariate analysis between colorectal schwannomas and GISTs.

		Schwannomas (n = 13)	GISTs (n = 21)	P Value
Age (mean ± standard deviation, years)		61.8 ± 11.1	60.5 ± 9.7	0.576
Sex (M:F)		7:6	15:6	0.249
Size (mean ± standard deviation, cm)		2.4 ± 1.2	6.3 ± 4.5	***0*.*002***
Longitudinal location	non-rectum	10 (76.9)	2 (9.5)	***<0*.*0001***
	rectum	3 (23.1)	19 (90.5)	
Transverse location	endophytic or dumbbell	9 (69.2)	9 (42.9)	0.134
	exophytic	4 (30.8)	12 (57.1)	
Shape	round	11 (84.6)	11 (52.4)	0.059
	oval	2 (15.4)	10 (47.6)	
Margin	smooth	11 (84.6)	10 (47.6)	***0*.*034***
	lobulated	2 (15.4)	11 (52.4)	
Homogeneity	homogeneous	12 (92.3)	11 (52.4)	***0*.*017***
	heterogeneous	1 (7.7)	10 (47.6)	
Necrosis	absent	12 (92.3)	11 (52.4)	***0*.*017***
	present	1 (7.7)	10 (47.6)	
Surface ulceration	absent	13 (100)	18 (85.7)	0.222
	present	0 (0)	3 (14.3)	
Calcification	absent	13 (100)	16 (76.2)	0.073
	present	0 (0)	5 (23.8)	
Attenuation on portal phase	iso	2 (15.4)	15 (71.4)	***0*.*002***
	high	11 (84.6)	6 (28.6)	
Lymph node enlargement	absent	9 (69.2)	21 (100)	***0*.*015***
	present	4 (30.8)	0 (0)	
Metastasis	absent	13 (100)	19 (90.5)	0.374
	present	0 (0)	2 (9.5)	

GIST = gastrointestinal stromal tumor, Numbers in parenthesis are percentage.

P values in ***Bold Italic*** indicate statistical significance.

Binary logistic regression analysis using a forward LR method showed that only the non-rectum location remained independent predictors for colorectal schwannomas differentiated from GISTs (odds ratio = 31.667; 95% confidence interval, 4.523–221.722; P = 0.001).

## Discussion

Study results showed that several CT features, i.e., smaller size, non-rectum location, smooth margin, homogeneously high attenuation without necrosis, and the presence of LN enlargement were statistically significant in the differentiation of colorectal schwannomas from GISTs. In addition, binary logistic regression analysis revealed that non-rectum location was the sole CT discriminator of schwannomas from GISTs (odds ratio = 31.667). The results were similar to previous reports. According to several publications by Miettinen et al., only 5% (1/20) colorectal schwannomas were located in the rectum, while 78.2% (133/170) of colorectal GISTs were located in the rectum, the third common site for GISTs following the stomach and small bowel [4, 8, 9]. The median size of the colorectal schwannomas was also reported to be 3 cm (range, 0.5–5 cm), while it was 6 cm (range, 0.5–15 cm) in colorectal GISTs [4, 8, 9]. Mean size (6.3 cm) of our GISTs was significantly larger than that (2.4 cm) of schwannomas (P = 0.001). When the cut-off value of tumor size was set at 3.9 cm in our study, AUC, sensitivity, and specificity were 0.808, 66.7% (14/21), and 92.3% (12/13), respectively. Therefore, we should pay attention to the possibility of GISTs because of their high malignant potential when we encounter a larger tumor (> 3.9 cm) located in the rectum.

Only one colorectal schwannoma showed heterogeneous enhancement with intra-tumoral necrosis in our study. The absence of necrosis in colorectal schwannomas is a well-known feature that has never been reported on imaging studies in the pathologic field [4]. This should not be surprising considering the relatively slow speed of tumor growth in benign tumors which is typically on par with that of neovascularization. Conversely, in malignant tumors such as GISTs, the speed of tumor growth often outstrips neovascularization and leads to central necrosis. Several previous reports indicate 37.5%-81.8% of rectal GISTs show heterogeneous enhancement with internal hemorrhage or necrosis [10, 11]. Indeed, half of GISTs (10/21, 47.6%) in our study had intra-tumoral necrosis. We believe that the results of this study suggest and confirm that the presence of necrosis is a differentiating CT feature of colorectal GISTs from schwannomas that have already been well-established in the field of pathology.

The frequency of LN enlargement is low; however, it was exclusively found in colorectal schwannomas (4/13, 30.8%). This result partly coincides with previous studies in which gastric schwannomas (75%-81.3%) more frequently accompany with enlarged LNs than gastric GISTs (5%-28.6%) [5, 12]. The incidence of enlarged LNs in previous studies was higher than in our study as they used a less strict size criteria for lymph node enlargement, i.e., 5 mm in short axis diameter in the previous study [12] versus 8 mm in long axis in our study. The larger tumor size (mean size ± standard deviation, 6.3 ± 1.8 cm) in the previous study might be also responsible for a higher incidence of enlarged LNs in the schwannoma group [5]. Pathophysiology of accompanying LN enlargement has not been clearly investigated; however, we hypothesize that lymphoid cuffing around the tumor, which is a characteristic histologic feature of gastrointestinal schwannomas, might be related to this phenomena. Several pathologists insist that lymphoid cuffing might be the result of cytokines systemically secreted by tumor cells that induce the chemokinesis of lymphocytes [1, 13]. Contrary to schwannoma, it is well-known that GISTs seldom accompany with LN metastasis [10, 11]. Therefore, schwannoma should be first considered when we encounter a subepithelial mass with enlarged LNs in the colorectum. This observation and hypothesis should be further investigated.

Contrary to our expectations and the previous report, the degree of enhancement is higher in colorectal schwannomas than in GISTs [5]. Most schwannomas (11/13, 84.6%) showed high attenuation than adjacent back muscle, while two thirds of GISTs (15/21, 71.4%) showed iso-attenuation. The degree of attenuation on portal phase was mostly iso or low attenuation in gastric lesions and was not significantly different between the two tumor groups [5]. We do not exactly know why colorectal schwannomas show high attenuation; however, a smaller size and non-rectum location of schwannoma might be responsible for the high enhancement.

There are several limitations in our study. First, statistical power may have been comparatively weak due to the relatively small sample size and inherent limitations of the retrospective nature of our study. However, our study should be considered useful as it is the first report systematically analyzing the differential CT features of colorectal schwannomas from GISTs. Second, CT protocols were not standardized because the patients with colorectal schwannomas were collected from four different hospitals. However, we believe the limitations on the use of different CT protocols may be insignificant because most CT scans were performed using MDCT scanners and with reconstruction interval and slice thicknesses that were less than or equal to 5 mm. Third, we did not analyze the diagnostic performance of the radiologists to differentiate between two tumor groups on CT. Finally, the results might be overestimated because we did not include other subepithelial tumors such as leiomyoma or neuroendocrine tumor. Further studies recruiting various kinds of colorectal subepithelial neoplasms are strongly warranted.

In conclusion, colorectal schwannomas are usually located in non-rectum and appear as small subepithelial nodules showing homogeneous high attenuation without necrosis and a smooth margin. Schwannomas exclusively accompany with enlarged lymph nodes. Using these CT findings, colorectal schwannomas can be differentiated from GISTs with high diagnostic performance.

## Supporting Information

S1 File (XLSX)

## Abbreviations

GIT: gastrointestinal tract
GIST: gastrointestinal stromal tumor
LN: lymph node
